# Natural killer cells regulate Th1/Treg and Th17/Treg balance in chlamydial lung infection

**DOI:** 10.1111/jcmm.12821

**Published:** 2016-03-29

**Authors:** Jing Li, Xiaojing Dong, Lei Zhao, Xiao Wang, Yan Wang, Xi Yang, Hong Wang, Weiming Zhao

**Affiliations:** ^1^ Department of Pathogenic BiologyShandong University School of MedicineJinanShandongChina; ^2^ Institute of Basic Medical ScienceQilu Hospital of Shandong UniversityJinanShandongChina; ^3^ Department of PathologyShandong University School of MedicineJinanShandongChina; ^4^ Department of Immunology and Department of Medical MicrobiologyFaculty of MedicineUniversity of ManitobaWinnipegMBCanada

**Keywords:** *Chlamydia*, natural killer cells, Th1/Treg, Th17/Treg, immunoregulation

## Abstract

Natural killer (NK) cell is an important component in innate immunity, playing a critical role in bridging innate and adaptive immunity by modulating the function of other immune cells including T cells. In this study, we focused on the role of NK cells in regulating Th1/Treg and Th17/Treg balance during chlamydial lung infection. We found that NK cell‐depleted mice showed decreased Th1 and Th17 cells, which was correlated with reduced interferon‐γ, interleukin (IL)‐12, IL‐17 and IL‐22 production as well as T‐bet and receptor‐related orphan receptor gamma t expression compared with mice treated with the isotype control antibody. In contrast, NK cell depletion significantly increased Treg in cell number and related transcription factor (Foxp3) expression. The opposite trends of changes of Th1/Th17 and Treg led to significant reduction in the Th1/Treg and Th17/Treg ratios. The data implicate that NK cells play an important role in host defence against chlamydial lung infection, mainly through maintaining Th1/Treg and Th17/Treg balance.

## Introduction

*Chlamydia* agents are obligate intracellular parasites of mammalian cells that cause myriad severe diseases 1, 2, 3. Infection of mice with a *Chlamydia trachomatis* (*C. trachomatis*) mouse pneumonitis strain (now classified as a new species, *C. muridarum*) through pulmonary or genital tract routes has been extensively used to study the immunobiology of *Chlamydia* infection. Data from mouse models and clinical studies have demonstrated that CD4^+^T cells expressing interferon γ (IFN‐γ; Th1) is the main immune component providing host protection against *C. trachomatis* 4, 5, 6.

Natural killer (NK) cells are large granular lymphocytes that are a first line of host defence against a wide range of microbial pathogens. Through cytotoxic activity and cytokine production, NK cells can kill infected cells without prior sensitization 7, 8, 9. The effect of NK cells on the host response has been studied in chlamydial infection, with inconsistent findings on the role of NK cells in different chlamydial infection models 10, 11, 12. Recent study showed that NK cell‐depleted mice showed exacerbated disease, decreased Th1 cellular response in the spleen and less mature dendritic cell (DC) phenotype during respiratory‐tract chlamydial infection, suggesting an important role of NK cells in the development of protective type 1 T‐cell immune responses against chlamydial infection by modulating DC function 13. However, the study of immunoregulatory effect of NK cells on other aspects of the adaptive immune response during chlamydial infection remains limited. Th1, Th17 cells and regulatory T cells (Treg) are distinct T‐cell subsets involved in chlamydial infection. Th1 cells have been well documented as the major protective factors in the resolution of chlamydial infection mainly through producing IFN‐γ 6; Th17 cells express retinoid‐acid receptor‐related orphan receptor gamma t (RORγt) and producing multiple pro‐inflammatory cytokines such as interleukin 17 (IL‐17) and IL‐22 14. Treg expressing Foxp3 have an anti‐inflammatory property cell–cell contact‐dependent suppression or releasing anti‐inflammatory cytokines 15. Numerous studies have demonstrated the critical importance of Th1 and Th17 in protective immunity against chlamydial lung infection 4, 5, 6, 16, 17, 18. IL‐17/Th17 can promote type 1 T‐cell‐dependent protective immunity in respiratory‐tract *C. muridarum* infection 17. Treg have also been identified in local tissues in humans and mice with chlamydial infection. Importantly, recent study has suggested a role of Treg in tissue pathology during chlamydial infection 19, 20, 21, 22.

Growing evidence indicates suggest that NK cells can modulate Th1, Th17 cell and Treg responses in infections and inflammatory diseases 23, 24, 25, 26, 27, 28. Notably, the reported studies on the role of NK cell in modulating T‐cell subset are mainly restricted to particular organs such as spleen or mediastinal lymph node 13, 29. In particular, the effect of NK cell on Treg has not been addressed in chlamydial infection. Therefore, a more comprehensive study on T‐cell subsets in spleen, infection site (lung) and mediastinal lymph nodes is need. In the present study, we aimed to evaluate the role of NK cells in the development of the T‐cell response, especially Th1 and Th17 as well as Treg responses during chlamydial lung infection. We used a NK cell‐specific antibody, anti‐asialo GM1, which has been commonly used as one of the most precise tools available to specifically eliminate NK cells 30, 31, 32 and compared the NK‐depleted mice with mice treated with isotype control antibody in protection and T‐cell responses in chlamydial lung infection. We confirmed the previous report showing that NK cell depletion induced significant decrease in protective Th1 and Th17. More importantly, we found that NK cell depletion significantly increased Treg response, leading to imbalanced Th1/Treg and Th17/Treg responses. Thus, the current study implicates a critical role of NK cells in the host defence against chlamydial lung infection by maintaining Th1/Treg and Th17/Treg balance.

## Materials and methods

### Mice

Male BALB/c mice (6–8 weeks old) were purchased from Vital River Laboratories (Beijing, China). The mice were housed in a specific pathogen‐free laminar flow cabinet. All animal experiments were conducted in compliance with the guidelines issued by the China Council for Animal Care and Utilization Committee of Shandong University, China (Permit Number: MECSDUMS2012056). The investigation conforms to the US National Institutes of Health Guide for the Care and Use of Laboratory Animals and was performed in accordance with the ARRIVE guidelines (http://www.nc3rs.org/ARRIVE).

### *Chlamydia*

*Chlamydia muridarum* organisms (Nigg strain) were cultivated, purified and quantified as described 33. The purified EBs were suspended in SPG buffer and stored at −80°C. The same seed stock of EBs was used throughout this study.

### NK cell depletion *in vivo*

We used polyclonal anti‐asialo‐gangli‐N‐tetraosylcer‐amide antibody (anti‐asialo GM1) (Wako, Osaka, Japan) to deplete NK cells in all experiments 34. Mice received an intravenous tail‐vein injection of 20 μl anti‐asialo GM1 or control normal rabbit IgG antibody (isotype) in 50 μl PBS 1 day before and 1 day after *C. muridarum* infection, then every 3 or 5 days injected with 10 μl anti‐asialo GM1 or isotype in 50 μl PBS until the end of the test.

### Mice infection and quantification of *in vivo* bacterial load

For mouse infection, 1 × 10^3^ inclusion‐forming units (IFUs) of live *C. muridarum* organisms in 40 μl SPG buffer were used to inoculate mice intranasally. Body weights of mice were monitored daily. At predetermined days after inoculation, the mice were killed under light anaesthesia with isoflurane and the lungs were aseptically isolated. The lung tissues were homogenated by using a glass homogenizer in 2 ml cold SPG buffer. The lung homogenates were centrifuged at 1600 g. for 30 min. at 4°C, and supernatants were stored at −80°C after split charging. The lung *C. muridarum* burden was assessed by infection of Hep‐2 cells and immunostaining of chlamydial inclusions.

### Histology

Lungs from different group of mice were removed aseptically at various times postinfectionand fixed in 4% paraformaldehyde solution for 24 hrs, and then embedded in paraffin for histology as described 35. The tissue sections (4‐μm thick) were stained with haematoxylin & eosin. Histopathology was scored according to a set of custom designed criteria 36. Changes were determined by light microscopy under a microscope at ×200 magnification.

### Lung mononuclear cells, splenocytes, mediastinal lymph node cells preparation, culture and cytokine measurements

To obtain single lung cell suspensions, lungs were cut into small pieces and then digested for 1 hr at 37°C with 1 mg/ml collagenase XI (Sigma‐Aldrich, St. Louis, MO, USA) in 3 ml RPMI 1640 and 300 mM EDTA, then the tissue fragments were blown into suspension and centrifuged at 300 g. for 8 min. The cell pellet was resuspended in 5 ml 35% (v/v) Percoll (Solarbio, Beijing, China) and centrifuged at 700 g. for 12 min. at room temperature. Red blood cells were lysed with ACK Lysing buffer followed by two washes in RPMI 1640 with 2% newborn bovine serum. After cell counting, single‐cell suspensions from lung mononuclear cells were cultured at 5 × 10^6^ cell/ml. Spleens and mediastinal lymph nodes were aseptically removed. Single‐cell suspensions from spleens and mediastinal lymph nodes were cultured at 7.5 × 10^6^ and 5 × 10^6^ cell/ml respectively. All cells were cultured with heat‐inactivated (65°C, 30 min.) *C. muridarum* (10^5^ IFU/ml) in complete culture medium (RPMI 1640 containing 10% heat‐inactivated FBS, 25 mg/ml gentamicin, 2 mM L‐glutamine and 0.05 mM 2‐mercaptoethanol). The supernatant was harvested after 72‐hr culture, and the production of cytokines [IFN‐γ, IL‐17A, IL‐22, IL‐10, IL‐6 and transforming growth factor β (TGF‐β)] was measured by ELISA (eBioscience, SanDiego, CA, USA). Cytokines (IFN‐γ, IL‐22) levels in serum were assayed by ELISA.

### Flow cytometry

To examine the expansion of NK cells and Treg after *C. muridarum* infection, freshly isolated splenocytes and lung mononuclear cells from anti‐asialo GM1 and isotype antibody‐treated mice were stained with anti‐CD3ε and anti‐49b (DX5) antibodies (eBioscience) to examine NK cell proportion. Antibodies specific for Foxp3 (intracellular staining), CD3e, CD4, CD25 (all antibodies were purchased from eBioscience) were used to analyse the proportion of Treg in splenocytes, lung mononuclear cells and mediastinal lymph nodes cells. Analyses involved use of the FACS Calibur flow cytometer (BD Biosciences, SanDiego, CA, USA) and data were analysed by using FCS express software.

### Intracellular cytokine staining

Splenocytes, lung mononuclear cells and mediastinal lymph node cells were incubated at 2 × 10^6^ cells/ml in 48‐well plates, and stimulated with cell stimulation cocktail (2 μl/well; eBioscience) for 2 hrs, then 1 μl Brefeldin A (eBioscience) was added for another 4 hrs. After washing with FACS buffer (PBS, 2% heat‐inactivated FBS, 0.09% sodium azide), cells were stained for surface markers with anti‐CD4 and anti‐CD3ε antibodies (eBioscience) for 30 min. at 4°C, then fixed and permeabilized with IC fixation buffer and permeabilization buffer (eBioscience) for 30 min. at 4°C and incubated with anti‐IFN‐γ or anti‐IL‐17a antibodies (eBioscience) for 1 hr at 4°C. The raw sample data were collected by using a FACS Calibur flow cytometer (BD Biosciences), and data were analysed by using FCS express software, all antibodies used in FCS staining were list in Figure S1.

### Quantitative PCR

RNA was extracted from mouse splenocytes, lung mononuclear cells and mediastinal lymph node cells by using Trizol reagent (Invitrogen, Carlsbad, CA, USA), and complementary DNA was synthesized by using the Primescript RT reagent kit with gDNA Eraser (Takara, Shiga, Japan). Quantitative PCR (Q‐PCR) involved use of the SYB green qPCR Mix (Takara) in a CFX96 real‐time PCR instrument (Bio‐Rad, Hercules, CA, USA). The sequences of primers used for Q‐PCR amplification were as follows: mouse RORγT forward/reverse 5′‐TCCACTACGGGGTTATCACCT‐3′/5′‐AGTAGGCCAC ATTACACTGCT‐3′, mouse Foxp3 forward/reverse 5′‐CACCTATGCCACCCTTATCCG‐3′/5′‐CATGCGAGTAAACCAATGGTAGA‐3′, mouse GATA3 forward/reverse 5′‐AAGCTCAGTATCCGCTGACG‐3′/5′‐GTTTCCGTAGTAGGACGGGAC‐3′ and mouse GAPDH forward/reverse 5′‐AGGT CGGTGTGAACGGATTTG‐3′/5′‐TGTAGACCATGTAG TTGAGGTCA‐3′. The results were normalized to GAPDH level. The mRNA levels were determined by determining the threshold cycle (Ct) of the target gene after normalization against the Ct value of GAPDH and calculated as 2 − [(Ct of target gene‐Ct of GAPDH) − Ct of control].

### Statistical analysis

All experiments were independently repeated at least three times. Statistical analysis involved use of Graphpad Prism 5 (Graphpad Software, San Diego, CA, USA). Data are presented as mean ± SEM. Comparisons between two groups involved unpaired Student's *t*‐test. *P* < 0.05 was considered statistically significant.

## Results

### NK cell‐depleted mice show more severe disease to chlamydial lung infection

**Figure 1:**
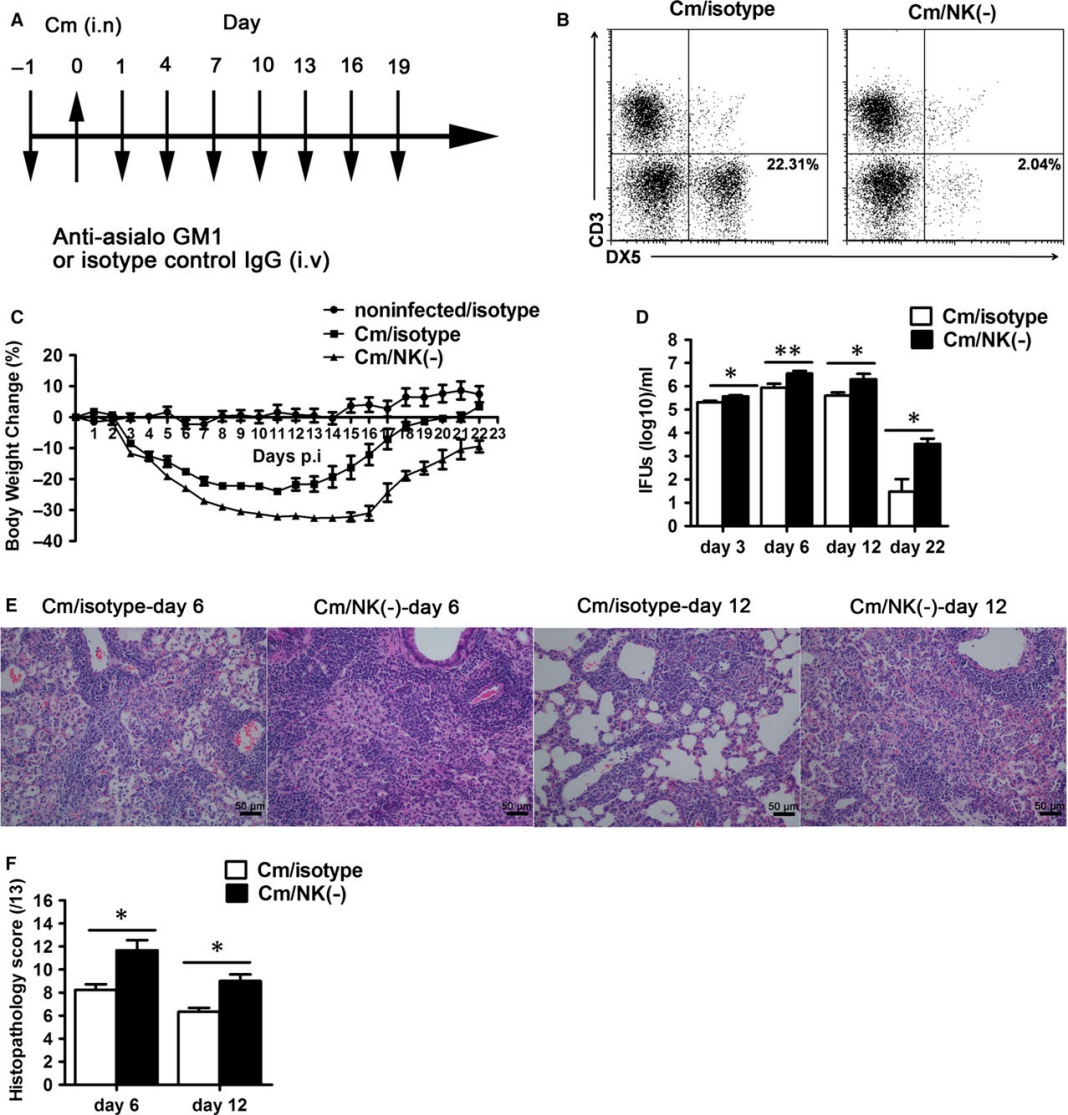
NK cell‐depleted mice show more severe disease following *Chlamydia muridarum* lung infection. BALB/c mice received tail‐vein injection (i.v) with 20 μl anti‐asialo GM1 [Cm/NK (‐)] or control normal rabbit IgG (Cm/isotype) antibody in 50 μl PBS 1 day before and 1 day after intranasal infection (i.n) with *C. muridarum* (1 × 10^3^ IFU), then every 3 or 5 days were injected with 10 μl anti‐asialo GM1 or isotype until the end of the test. The uninfected control mice were administered with isotype control antibody (noninfected/isotype) following the same schedule. (**A**) The schematic of NK cell deleption method. (**B**) Flow cytometric images of NK cell (CD3^−^ DX5^+^) in lung mononuclear cells from mice treated with anti–asialo GM1 or isotype antibody. (**C**) The bw changes in the noninfected/isotype, Cm/isotype and Cm/NK (‐) groups. (**D**) Mice were killed at day 3, day 6, day12 and day 22 after infection, and the lung tissues were homogenized and live chlamydial loads were measured as described in Materials and methods. (**E**) The lung tissue sections were stained with haematoxylin & eosin for histological analysis under light microscopy at ×200 magnification. (**F**) The score of tissue inflammatory grades were analysed as described in Materials and methods. At least three independent experiments with 4–5 mice in each group were performed, with one representative experiment is shown. Data are shown as mean ± S.E.M. *P < 0.05, **P < 0.01 *versus* isotype control.

Previous study has shown a protective role of NK cell in host defence chlamydial lung infection in C57BL/6 mice 13. To assess the role of NK cells in BALB/c mice, we intravenously injected anti‐asialo GM1 antibody (Fig. 1A) and found this treatment effectively depleted NK cells (Fig. 1B). Following chlamydial lung infection, NK cell‐depleted mice showed greater bw loss than control antibody‐treated mice and slower recovery (Fig. 1C). Even in the very early stage (day 3) when the changes in bw were similar between the two groups, the chlamydial loads (IFUs) in the lung were significantly greater in NK cell‐depleted mice than control mice (*P* < 0.05). The difference in chlamydial loads was still obvious at later stages (days 6 and 12,) and recovery stage (days 22, Fig. 1D). Consistently, histology analysis revealed more intense pathologic changes in NK cell‐depleted mice than isotype control mice (Fig. 1E and F).

### NK cell‐depleted mice exhibit decreased type 1 immune response to chlamydial lung infection

**Figure 2:**
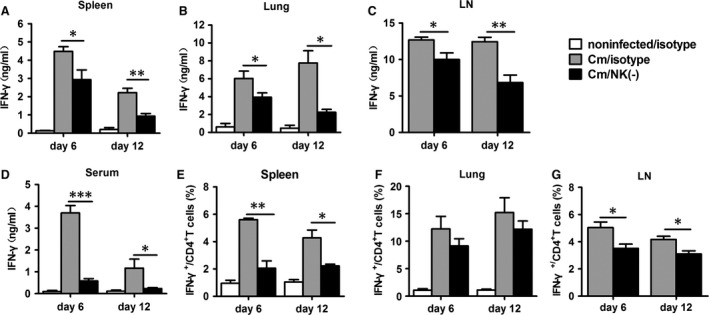
Decreased type 1 immune response in NK cell‐depleted mice following *Chlamydia muridarum* lung infection. BALB/c mice were treated with anti‐asialo GM1 or isotype antibodies and infected with *C. muridarum*as described in the legend to Figure 1, and killed at day 6 or day 12 after infection. (**A**–**C**) ELISA of IFN‐γ protein expression in splenocytes, lung mononuclear cells and mediastinal lymph node cells (LN) cultured with heat‐inactivated *C. muridarum* EBs (10^5^ IFU/ml) for 72 hrs. (**D**) ELISA of IFN‐γ production in serum. (**E**–**G**) Summary of intracellular cytokine staining data of IFN‐γ‐producing CD4^+^ T cells in the different organs. At least three independent experiments with 4–5 mice in each group were performed, with one representative experiment is shown. Data are shown as mean ± S.E.M. *P < 0.05, **P < 0.01, ***P < 0.001 *versus* isotype control.

Th1 immune response is the main immune component providing host protection against *C. trachomatis*, thus we examined the modulatory role of NK cells on Th1 immune response in local (lung), secondary lymphoid organs (spleen and mediastinal lymph node) and the circulatory system (serum). Cytokine analysis showed that NK cell‐depleted mice exhibited significantly lower IFN‐γ level in the spleen, lung, mediastinal lymph node and serum at days 6 and 12 compared with control mice (both *P* < 0.05, Fig. 2A–D). The frequency of IFN‐γ^+^ CD4^+^ T cells was lower in spleen and mediastinal lymph nodes of NK cell‐depleted than control mice at days 6 and 12 (Fig. 2E–G and Fig. S2). In lung, we found a significantly decreased total IFN‐γ production; however, the reduction in IFN‐γ^+^ CD4^+^ T cells was not so much as those in spleen and mediastinal lymph node after NK‐cell depletion. Previous study have reported NK cells are an important source of IFN‐γ and make up 10% of resident lymphocytes in lung 37, therefore, it is likely the depleted NK cells together with reduced IFN‐γ^+^ CD4^+^ T cells resulted in significant IFN‐γ reduction in lung of NK cell‐depleted mice. The results confirmed the significant modulating effect of NK cell on Th1 responses and protection during chlamydial lung infection in BALB/c mice.

### NK cell‐depleted mice show reduced IL‐17 and IL‐22 level with chlamydial lung infection

**Figure 3:**
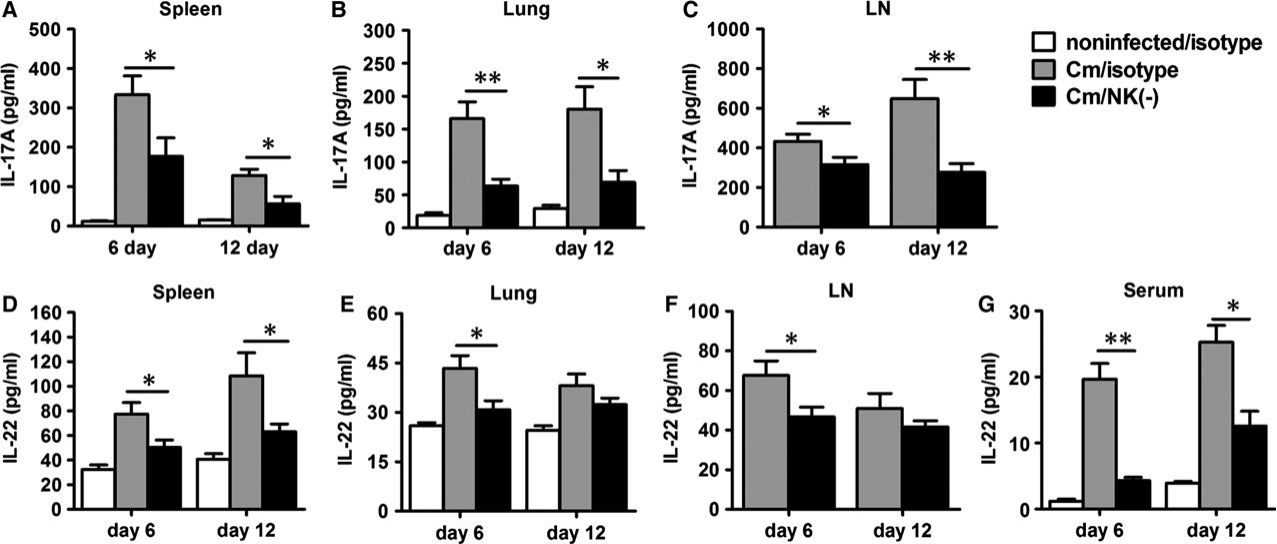
Reduced IL‐17 and IL‐22 expression in NK cell‐depleted mice with chlamydial lung infection. Mice were treated as described in the legend to Figure 1, and killed at day 6 or day 12 after infection, IL‐17A (**A**–**C**) and IL‐22 protein levels in 72 hrs culture supermants of splenocytes, lung mononuclear cells, mediastinal lymph node cells (**D**–**F**) and in serum (**G**) were determined by ELISA. At least 3 independent experiments with 4–5 mice in each group were performed, with one representative experiment is shown. Data are shown as mean ± S.E.M. *P < 0.05, **P < 0.01.

Because IL‐17 is also an important protective cytokine in the host defence against chlamydial lung infection 16, 17, we further investigated whether NK‐cell depletion affected the IL‐17/Th17 response. The results showed that NK cell‐depleted mice exhibited significantly lower production of IL‐17 by spleen, lung and mediastinal lymph node cells at days 6 and 12 postinfection (Fig. 3A–C). IL‐22 has been suggested to cooperate with IL‐17 in the host defence against chlamydial infection 38, so we examined the change in IL‐22 production after NK‐cell depletion and found that the level of IL‐22 was significantly lower in NK cell–depleted mice than control mice by splenocytes, lung mononuclear cells, mediastinal lymph node cells and serum (Fig. 3D–G).

### NK cell depletion reduces Th17 response in chlamydial lung infection

**Figure 4:**
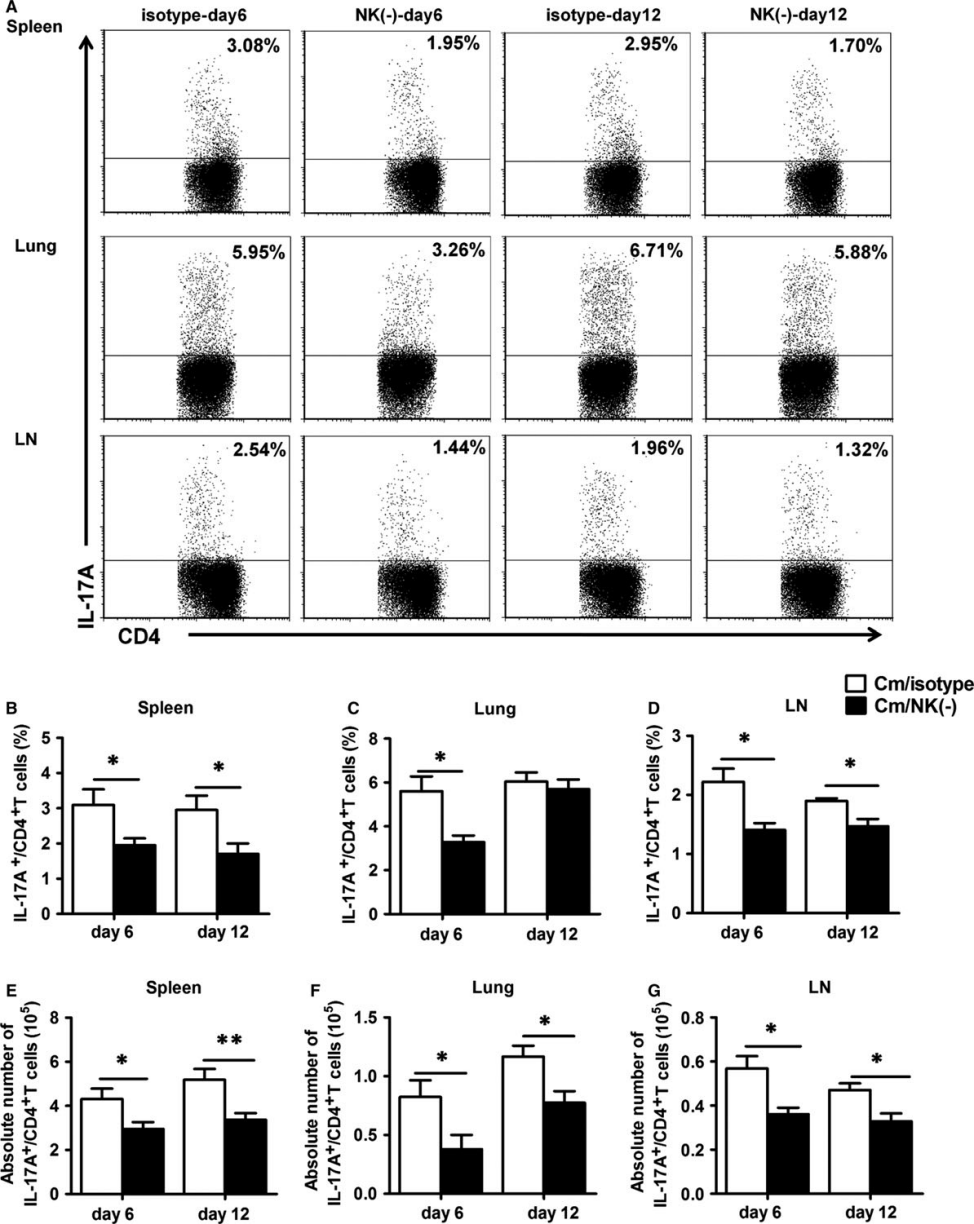
Reduction in IL‐17^+^ CD4^+^T cells response in NK cell‐depleted mice during chlamydial lung infection. Splenocytes, lung mononuclear cells and mediastinal lymph node cells from each group were prepared at days 6 or 12 after infection.IL‐17‐producing CD4^+^ T cells were quantified by intracellular cytokine staining as described in Materials and methods. (**A**) Representative flow cytometric images of IL‐17‐producing CD4^+^ T cells. Summary of the percentage (**B**–**D**) and absolute number (**E**–**G**) of IL‐17‐producing CD4^+^ T cells. At least three independent experiments with 4–5 mice in each group were performed, and one representative experiment is shown. Data are shown as mean ± S.E.M. *P < 0.05, **P < 0.01.

Although several other cell types such as γδT cells, ILC3s may be contribute to the production of IL‐17, and IL‐22, Th17 cells are the dominant source of these cytokines. So we did intracellular cytokine staining to confirm the effect of NK cell on Th17 cells in chlamydial lung infection. As shown in Figure 4A, intracellular cytokine staining and flow cytometric analysis demonstrated a significant decrease in the proportion and absolute number of IL‐17^+^ CD4^+^ T cells in NK cell‐depleted mice compared with control mice in spleen, lung and mediastinal lymph node cells on day 6 and 12 after infection (Fig. 4B–G). The results confirm a promoting role of NK cells on Th17 cell response in local infection site and secondary immune organs during chlamydial lung infection.

### NK‐cell depletion increases the production of CD4^+^ CD25^+^ Foxp3^+^ regulatory T cells

**Figure 5:**
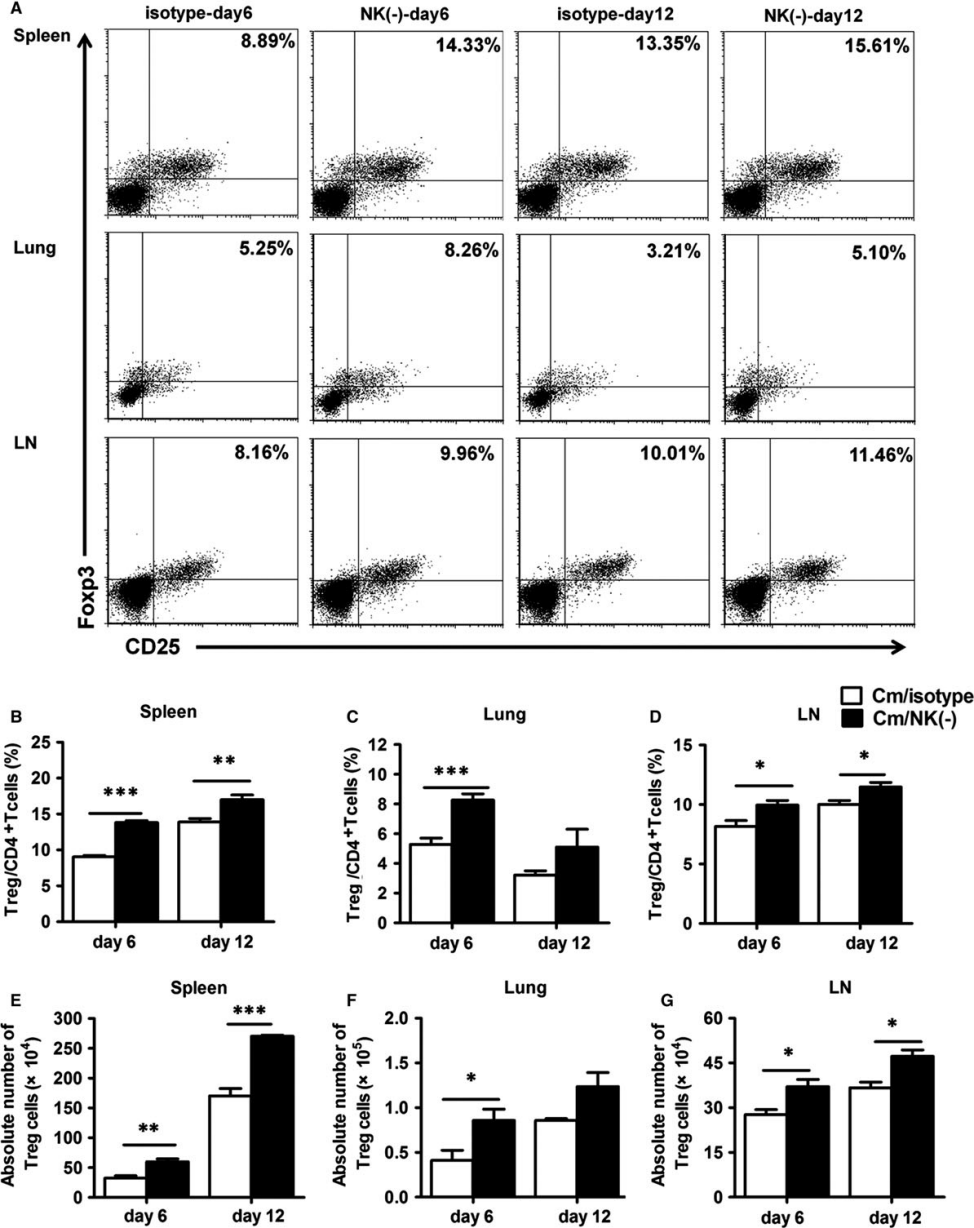
Increased CD4^+^ CD25^+^ Foxp3^+^ regulatory T‐cell responses in NK cell‐depleted mice. Splenocytes, lung mononuclear cells and mediastinal lymph node cells were isolated at day 6 or day 12 after *Chlamydia muridarum* infection. (**A**) Representative flow cytometric images of CD4^+^ CD25^+^ Foxp3^+^ T cells in the different organs. Summary of the percentage (**B**–**D**) and absolute number (**E**–**G**) of CD4^+^ CD25^+^ Foxp3^+^ T cells. At least three independent experiments with 4–5 mice in each group were performed, with one representative experiment is shown. Data are mean ± S.E.M. *P < 0.05, **P < 0.01, ***P < 0.001.

Considering the supressive role of Treg on immune responses of other cells 39, we further examined CD4^+^ CD25^+^ Foxp3^+^ T cells in the spleen, lung and mediastinal lymph node cells in NK cell‐depleted and control mice (Fig. 5A and Fig. S2). The proportion and number of CD4^+^ CD25^+^ Foxp3^+^ T cells was significantly higher in NK cell‐depleted mice than control mice at day 6 and 12 after infection (Fig. 5B). In particular, NK‐cell depletion also enhanced the proportion of CD4^+^ CD25^+^ Foxp3^+^ T cells in infection sites (lung tissues) and mediastinal lymph node cells at day 6 after infection (Fig. 5C, D and F, G). The results show an inhibitory role of NK on Treg responses especially at early stages of infection (day 6). Furthermore, IL‐10 is an important immunoregulatory cytokine and Treg is one of the main sources of this cytokine, so we measured IL‐10 levels in spleen, lung and mediastinal lymph nodes and found that IL‐10 production has no significant difference between isotype and NK cell‐depleted mice in our current model (Fig. S3).

### NK cell‐depleted mice show decreased T‐bet, RORγT expression, but increased Foxp3 expression during chlamydial lung infection

**Figure 6:**
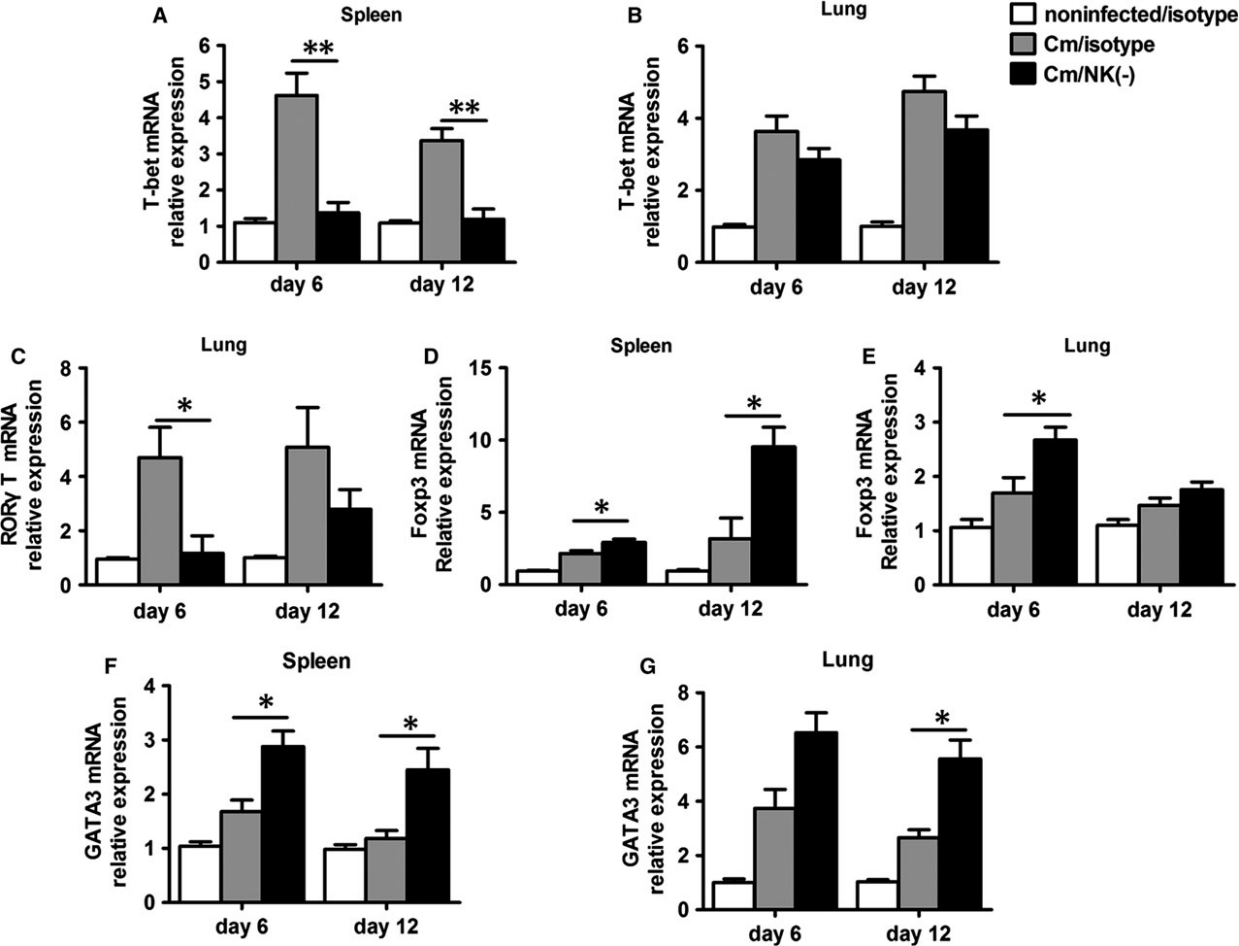
NK‐cell depletion decreases Th1, Th17 transcription factor (T‐bet, RORγT) but increases Treg and Th2 transcription factor (Foxp3,GATA3). Mice were treated and infected as described in the legend to Figure 1, and killed at day 6 or day 12 after infection. Total RNA extracted from the splenocytes and lung mononuclear cells which were cultured with heat‐inactivated *Chlamydia muridarum* (10^5^ IFU/ml) for 72 hrs, and transcription factors mRNA expression were measured by Q‐PCR using specific primers. Q‐PCR analysis of T‐bet in splenocytes and lung mononuclear cells (**A** and **B**), RORγT in lung mononuclear cells (**C**) and Foxp3,GATA3 in splenocytes and lung mononuclear cells (**D**–**G**) of NK cell‐depleted mice, isotype control and noninfected mice. At least three independent experiments with 4–5 mice in each group were performed, with one representative experiment is shown. Data are shown as mean ± S.E.M. *P < 0.05 , **P < 0.01.

To confirm the findings of opposite trend in Th1/Th17 and Treg responses in NK‐depleted mice (Figs. 2, 3, 4, 5), we further compared the mRNA expression of T‐bet, RORγT and Foxp3, the specific transcription factor for Th1, Th17 and Treg respectively. As expected, the mRNA levels of T‐bet and RORγt was significantly lower in NK cell‐depleted mice than control mice (*P* < 0.05, Fig. 6A–C), although the direct effects of NK‐cell depletion may also partially contributed to the reduced T‐bet mRNA expression. A similar trend of the change in T‐bet and RORγt (day 12) expression in lung was observed, although the difference did not reach statistical significance (Fig. 6B and C). Even it can be also expressed by other non‐Th17 cells such as ILC3, splenic RORγt mRNA was still exhibited low level in all groups (data not shown). Consistent with findings in Figure 5, the mRNA level of Foxp3 was significantly higher in NK‐celldepleted mice than control mice in the spleen at both days 6 and 12 (Fig. 6D) and in the lung at day 6 (Fig. 6E). We also detected the level of GATA3 (Th2 transcription factor) mRNA expression in spleen and lung, and found it was significantly higher in NK cell‐depleted mice (Fig. 6F and G). The data further confirm the opposite modulating effect of NK cell on Th1/Th17 and Treg in the transcription level, T‐bet, RORγt and Foxp3.

### NK‐cell depletion decreases IL‐12p40, IL‐6 level and increases TGF‐β production

**Figure 7:**
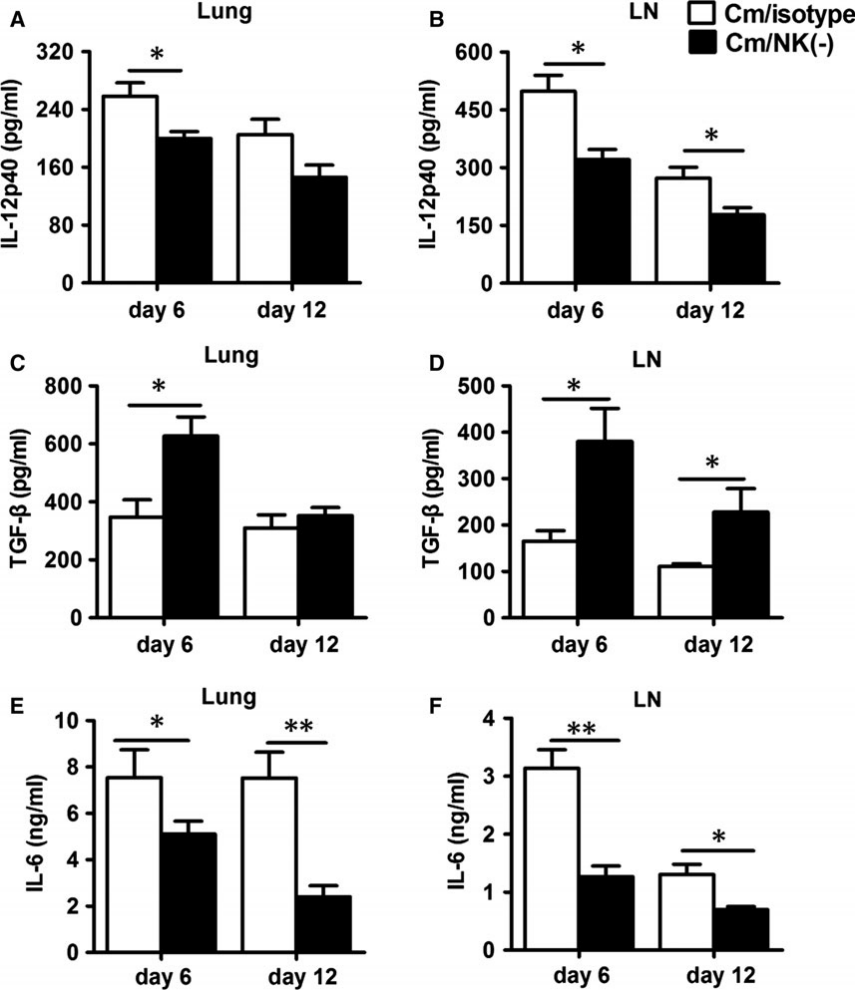
NK‐cell depletion increases TGF‐β production but decreases IL‐12p40 and IL‐6 production. ELISA of IL‐12p40 (**A** and **B**), TGF‐β (**C** and **D**) and IL‐6 (**E** and **F**) in lung mononuclear cells and mediastinal lymph node cells which were cultured with UV‐inactivated *Chlamydia muridarum* EBs for 72 hrs. At least three independent experiments with 4–5 mice in NK cell‐depleted group and isotype control group were performed, with one representative experiment is shown. Data are mean ± SEM. *P < 0.05, **P < 0.01.

To understand the molecular basis by which NK cells affect the ratio of Th17/Treg in chlamydial lung infection, we studied IL‐12p40, IL‐6 and TGF‐β levels in the NK cell‐depleted mice because these cytokines play important role in the differentiation of Th1, Th17 and Treg cells 40, 41. We found that IL‐12p40, the common chain for IL‐12 and IL‐23, was significantly lower in NK cell‐depleted mice than control mice (Fig. 7A and B). In addition, we found that TGF‐β levels was higher in NK cell‐depleted mice than control mice in the lung and mediastinal lymph node cells at days 6 and 12 after infection (Fig. 7C and D) while IL‐6 level was lower in NK cell‐depleted mice than control mice in both lung and mediastinal lymph node cells at days 6 and 12 (all *P* < 0.05, Fig. 7E and F). The alteration in IL‐12p40, IL‐6 and TGF‐β levels may contribute to the observed changes in Th1, Th17 and Treg responses.

### NK‐cell depletion leads to imbalanced Th1/Treg and Th17/Treg responses

**Figure 8:**
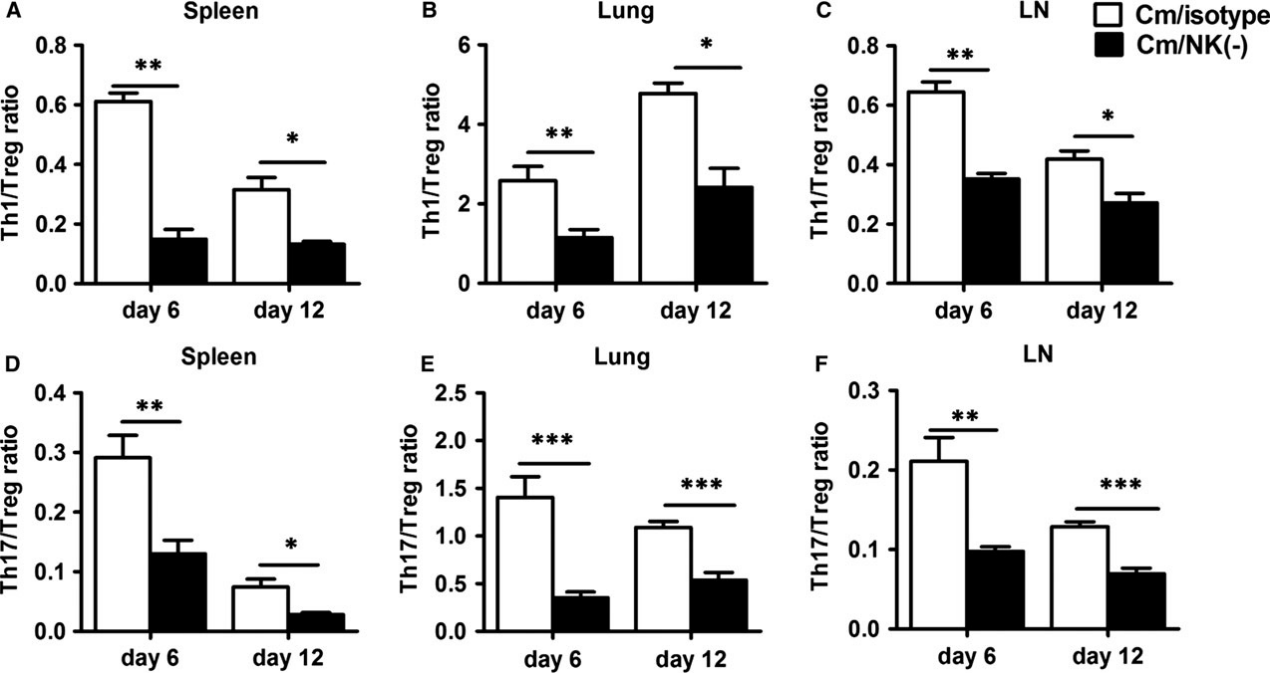
NK‐cell depletion reduces Th1/Treg and Th17/Treg ratio. Th1/Treg and Th17/Treg ratio in isotype control and NK cell‐depleted mice in splenocytes (**A** and **D**), lung mononuclear cells (**B** and **E**) and mediastinal lymph node cells (**C** and **F**) were calculated by the numbers of IFN‐γ‐produced CD4^+^ T cells or IL‐17‐produced CD4^+^ T cells by the number of CD4^+^ CD25^+^ Foxp3^+^ regulatory T cells, respectively. At least three independent experiments with 4–5 mice in each group were performed, with one representative experiment is shown. Data are mean ± S.E.M. *P < 0.05, **P < 0.01, ***P < 0.001.

Considering the possible opposite role of Th1/Th17 and Treg in host defence against chlamydial lung infection, we further analysed the ratio of Th1 and Th17 cells to Treg in NK cell‐depleted mice. Compared to the observed alteration in single parameters (Th1, Th17 or Treg), the changes in the ratios of Th1 or Th17 to Treg are more dramatic, showing the bias to Treg in NK‐depleted mice. As shown in Figure 8, at day 6 after infection, the isotype control antibody treated mice showed a significantly increased Th1/Treg and Th17/Treg ratios in comparison with uninfected mice (data not shown) both in the spleen and lung. However, the increased Th1/Treg or Th17/Treg ratio by infection was not observed in the NK cell‐depleted mice in the spleen, lung and mediastinal lymph nodes. The same is true at the later stage (day 12) although the levels were lower. Therefore, the depletion of NK cell lead to more biased response to Treg, thus imbalanced Th1/Treg and Th17/Treg responses during chlamydial lung infection.

## Discussion

In this study, we demonstrated that NK cells play an important immunomodulatory role in Th1, Th17 and Treg responses during chlamydial lung infection in BALB/c mice. Specifically, NK cell‐depleted mice showed increased susceptibility to *C. muridarum* lung infection and more serious pathology, which was associated with significantly decreased Th1 and Th17 response but increased Treg response. The results demonstrate a critical role of NK cell in maintaining the balance of Th1/Treg and Th17/Treg responses in chlamydial lung infection.

The promoting role of NK cells on splenic Th1 immune response has been reported in chlamydial lung infection in C57BL/6 mice 13. In this study, we extended the finding to BALB/c mice and lung Th1 cells and more importantly, we demonstrated that NK cells have a divergent role in affecting the immune responses of Th17 cells and Treg. The NK cell‐depleted mice showed a lower Th17 response as compared with control mice, as supported by a reduced absolute number and percentage of IL‐17^+^ CD4^+^ T cells (Fig. 4) and decreased production of IL‐17 and IL‐22 (Fig. 3). Furthermore, we found reduced mRNA level of RORγT, the key transcription factor for Th17‐cell differentiation in NK cell‐depleted mice. Considering the synergistic effect of Th1 and Th17 response in protection against chlamydial infection, the promoting effect of NK cells on both Th1 and Th17 responses is significant in host defence against the infection.

A novel finding in this study is the significantly enhanced Treg response in NK cell‐depleted mice during *C. muridarum* infection. Both the percentage and absolute number of CD4^+^ CD25^+^ Foxp3^+^ T cells in spleen, lung and mediastinal lymph node cells were significantly increased with NK‐cell depletion (Fig. 5), which was associated with increased mRNA expression of the specific transcription factor Foxp3. Treg play a significant role in tuning down the effector immune response and suppression of inflammatory responses, which can be both beneficial and deleterious in host defence. Recently, several studies have examined the role of Treg in chlamydial infection 20, 42. Depletion of Treg caused increased inflammatory T‐cell response 20. In addition, depletion of Treg increased Th1 response and reduced tissue pathology in the genital tract 42. Moreover, PC 61 treatment to deplete Treg before *C. muridarum* genital infection reduced oviduct pathology without altering the bacterial loads in the genital tract 21. In our study, after NK‐cell depletion, we found a significantly increased Treg response, together with decreased Th1 and Th17 response, which led to reduced Th1/Treg and Th17/Treg ratio. Although the mechanism by which NK cells inhibits Treg remains unclear, several studies have shed light on this. It has been reported that activated NK cells could inhibit the CD28‐mediated conversion of CD4^+^ CD25^−^ T lymphocytes into CD4^+^ CD25^+^ Treg, and IFN‐γ produced by NK cells could inhibit Treg conversion 26. In addition, activated NK cells can lyse expanded human Treg induced by co‐culture with *Mycobacterium tuberculosis*‐stimulated monocytes, which could be inhibited by blocking NKG2D or NCR1 27. Furthermore, it has been found that NK cells can modulate the function of DCs 43. In particular, the DCs from NK cell‐depleted mice produced higher IL‐10 and lower IL‐12p40. The altered cytokine pattern of DCs in NK‐depleted mice may contribute to the increase in Treg and reduction in Th1 and Th17 responses.

Because of the different roles played by Th1/Th17 cells and Treg in chlamydial infections, a balance of the these cell types particularly the ratio of protective cells (Th1 and Th17) and the pathological cells (Treg) can determine the outcome of chlamydial infections, fast recovery or persistent infection. In this study, we observed an imbalance between Treg and Th1, Th17 cells after NK cell depletion. Indeed, the significantly reduced Th1/Treg and Th17/Treg ratios in spleen, lung and mediastinal lymph nodes (Fig. 8) was correlated with more severe lung infection and pathology in NK cell‐depleted mice. Furthermore, the analysis of the local cytokine microenvironment (mediastinal lymph node and lung) suggested that the reduced Th17/Treg ratio in NK cell‐depleted mice was closely associated with significantly reduced IL‐12 and IL‐6 levels and increased TGF‐β production (Fig. 7). Both IL‐6 and TGF‐β are crucial for the balance of Th17 cells and Treg 44. TGF‐β together with IL‐6 can induce the expression of RORγT, which directs the differentiation of Th17 cells, whereas TGF‐β induces Foxp3 expression and Treg cell development 45. Furthermore, Foxp3 can inhibit RORγT‐induced Th17 cell differentiation 44. Therefore, the reduced IL‐6 production but increased TGF‐β production would prefer the development of Treg rather than Th17 cells with NK‐cell depletion.

Although the promoting effect of NK cells on Th1 response particularly on spleen cells has been reported 13, 46, the present comprehensive study on local (lung) and secondary lymphoid organs (spleen and mediastinal lymph node) provides stronger evidence for the critical of NK cells in bridging innate and adaptive immunity in chlamydial lung infection. In addition, the present study has demonstrated the role of NK cells in a different strain, BALB/c mice, instead of C57BL/6 mice 13. This point is important because these two mouse strains are reportedly different in susceptibility to chlamydial infections 47, 48. Based on the data from our study, NK cells play a consistent role in the tested different mouse strains, particularly promoting Th1 and Th17 response, but inhibiting Treg response.

In conclusion, NK cells play an important immunomodulatory role in the Th1, Th17 and Treg responses to chlamydial lung infection. It maintains the balance of Th1/Treg and Th17/Treg, thus helpful for bacteria clearance and pathology reduction. Our finding expands our understanding on the role of NK cells in protective immunity against chlamydial infections and provides new insights into the linkage between innate and adaptive immune responses in infectious diseases. Further studies are necessary to explore the mechanisms by which NK cells exert their function in modulating different T‐cell subsets. In addition, our current study only observed NK cells effect by administration NK‐cell depletion antibody before and during infection, further approaches such as adoptive transfer NK cells or administration depletion antibody after the initiation of infection will be more beneficial for using NK cells in developing prophylactic and/or therapeutic strategies for chlamydial diseases.

## Conflicts of interest

The authors disclose no potential conflicts of interest.

## Supporting information


**Figure S1** Details of antibodies used in Flow cytometric analysis.
**Figure S2** Flow cytometric gating strategies for different cell populations in the spleen.
**Figure S3** IL‐10 expression in NK cell‐depleted and isotype antibody‐treated mice during chlamydial lung infection.
