# Supplementary material for: Natural killer cells regulate Th1/Treg and Th17/Treg balance in chlamydial lung infection
Source: J Cell Mol Med. 2016 Mar 29;20(7):1339–51. doi: 10.1111/jcmm.12821 (PMC4929289; doi:10.1111/jcmm.12821)
Supplement: Supplementary file 1 — Figure S1 Details of antibodies used in Flow cytometric analysis. Figure S2 Flow cytometric gating strategies for different cell populations in the spleen. Figure S3 IL‐10 expression in NK cell‐depleted and isotype antibody‐treated mice during chlamydial lung infection. [file JCMM-20-1339-s001.docx]

Natural killer cells regulate Th1/Treg and Th17/Treg balance in chlamydial lung infection

Jing Li^a^, Xiaojing Dong^a^, Lei Zhao^b^, Xiao Wang^c^, Yan Wang^c^, Xi Yang^a,d^, Hong Wang^a,^*, WeimingZhao^a,^ *

^a^Department of Pathogenic Biology, Shandong University School of Medicine, Jinan, Shandong, China

^b^Institute of Basic Medical Science, Qilu Hospital of Shandong University, Jinan, Shandong, China

^c^Department of Pathology, Shandong University School of Medicine, Jinan, Shandong, China

^d^Department of Immunology and Department of Medical Microbiology, Faculty of Medicine, University of Manitoba, Winnipeg, Manitoba, Canada

*Correspondence to:

Weiming Zhao E-mail: [zhaowm@sdu.edu.cn](mailto:zhaowm@sdu.edu.cn)

Hong Wang E-mail: [hongwang@sdu.edu.cn](mailto:hongwang@sdu.edu.cn)

44 Wenhua Xi Road, Jinan, Shandong, 250012 P.R. China 0531-88382579

**Supplement data 1**

| **Table 1. Details of antibodies used in Flow cytometry** | | | |
| --- | --- | --- | --- |
| **Name** | **Clone** | **Species** | **Conjugate** |
| Anti-CD3e | 145-2C11 | mouse | APC |
| Anti-CD4 | GK1.5 | mouse | PE-Cyanine7 |
| Anti-IL-17 | eBio17B7 | mouse | PE |
| Anti-IFN-γ | XMG1.2 | mouse | PE |
| Anti-CD25 | PC61.5 | mouse | PE |
| Anti-Foxp3 | FJK-16s | mouse | PerCP-Cyanine5.5 |
| Anti-DX5(CD49b) | DX5 | mouse | FITC |


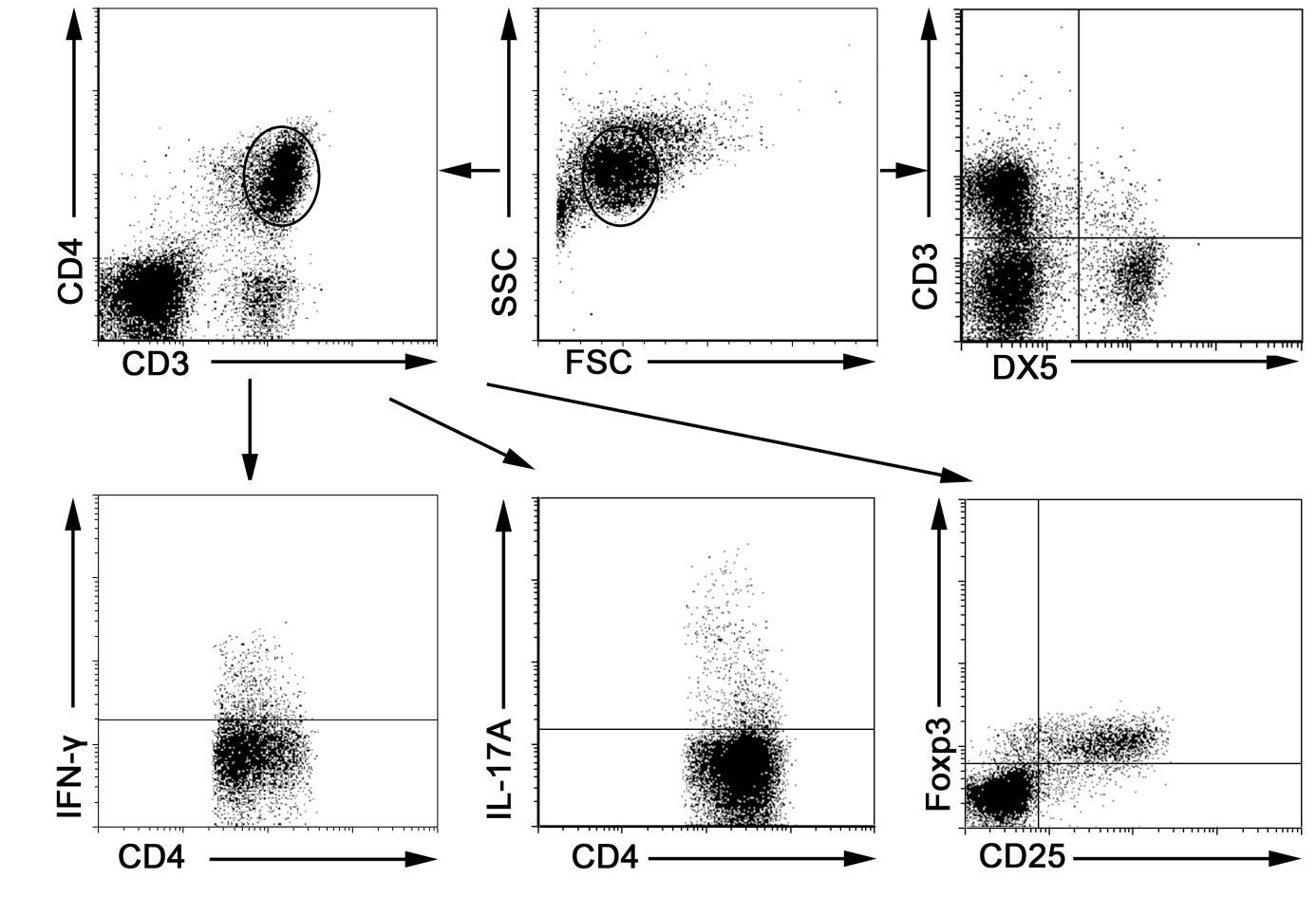


**Supplement data 2.** Flow cytometric gating strategies for different cell populations in the spleen. Splenocytes were isolated and stained with the following markers to differentiate different cell populations: CD3, DX5, CD4, CD25, IFN-γ, IL-17A, Foxp3. NK cell (CD3^-^DX5^+^), Th1 (CD3^+^CD4^+^IFN-γ^+^), Th17 (CD3^+^CD4^+^IL-17A^+^), Treg (CD3^+^CD4^+^CD25^+^ Foxp3^+^) were gated as shown in dot plot.


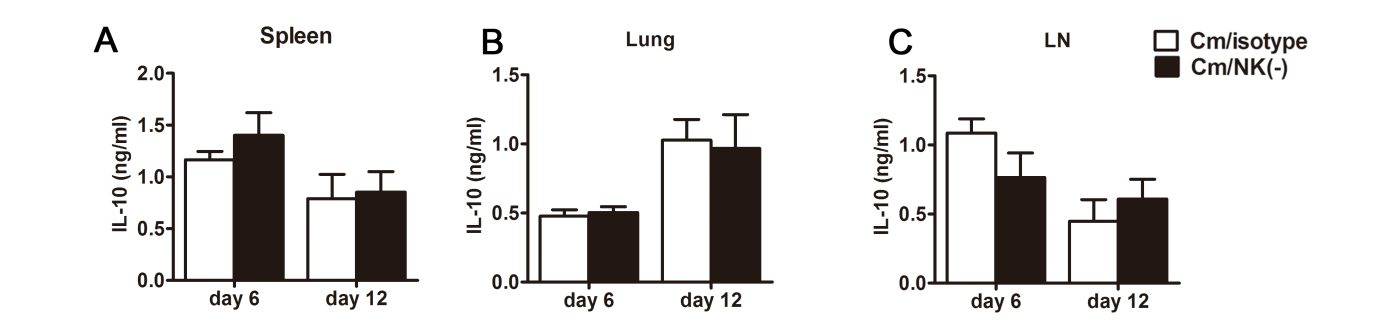


**Supplement data 3. IL-10 expression in NK cell-depleted and isotype antibody treated mice during chlamydial lung infection.** Mice were treated as method and material, and sacrificed at day 6 or day12 postinfection, IL-10 protein levels in 72 h culture supermants of splenocytes (A), lung mononuclear cells (B) and mediastinal lymph node cells (C) were determined by ELISA. At least 3 independent experiments with 4 to 5 mice in each group were performed, with one representative experiment shown. Data are shown as mean ± SEM.
